# Local application reduces number of needed EPC for beneficial effects on wound healing compared to systemic treatment in mice

**DOI:** 10.1007/s00068-021-01621-3

**Published:** 2021-04-04

**Authors:** Katharina Sommer, Heike Jakob, Tobias Kisch, Dirk Henrich, Ingo Marzi, Johannes Frank, Anna L. Sander

**Affiliations:** 1 Department of Trauma, Hand and Reconstructive Surgery, Hospital of the Johann Wolfgang Goethe-University, Theodor Stern Kai 7, 60590 Frankfurt am Main, Germany; 2 Department of Plastic and Hand Surgery, University of Lübeck, Lübeck, Germany

**Keywords:** Endothelial progenitor cells, Wound healing, Angiogenesis, Epithelialization

## Abstract

**Introduction:**

Stem cell transplantation is one of the most promising strategies to improve healing in chronic wounds as systemic administration of endothelial progenitor cells (EPC) enhances healing by promoting neovascularization and homing though a high amount of cells is needed. In the following study, we analysed whether local application can reduce the number of EPC needed achieving the same beneficial effect on wound healing.

**Material and Methods:**

Wound healing after local or systemic treatment with EPC was monitored in vivo by creating standardized wounds on the dorsum of hairless mice measuring wound closure every second day. Systemic group received 2 × 10^6^ EPC i.v. and locally treated group 2 × 10^5^ EPC, locally injected. As control PBS injection was performed the same way. Expression of CD31, VEGF, CD90 and, SDF-1α was analysed immunohistochemically for evaluation of neovascularisation and amelioration of homing.

**Results:**

Local (7.1 ± 0.45 SD) as well as systemic (6.1 ± 0.23 SD) EPC transplantation led to a significant acceleration of wound closure compared to controls (PBS local: 9.7 ± 0.5 SD, PBS systemic 10.9 ± 0.38 SD). Systemic application enhanced CD31 expression on day 6 after wounding and local EPC on 6 and 9 in comparison to control. VEGF expression was not significantly affected. Systemic and local EPC treatment resulted in a significantly enhanced SDF-1α and CD90 expression on all days investigated.

**Conclusion:**

Local as well as systemic EPC treatment enhances wound healing. Moreover, beneficial effects are obtained with a tenfold decrease number of EPC when applied locally. Thus, local EPC treatment might be more convenient way to enhance wound healing as number of progenitor cells is limited.

## Introduction

Wound healing is a complex process that requires a well-orchestrated interplay between different tissue structures and a large number of resident and infiltrating cell types. Especially, angiogenesis is an essential step in successful wound healing [1]. Progenitor cells take part in this orchestration, as they are able to regulate neovascularization and enhance healing by cytokine production [2]. Thus, it has already been shown that augmentation of local number of progenitor cells ameliorates impaired wound healing [3–5].

One fraction of progenitor cells are endothelial progenitor cells (EPC). EPC arise from the bone marrow and circulate in the blood [6]. These cells can be identified by uptake of DiLDL after cultivation [7]. Peripheral injected DiLDL-labeled EPC have been shown to migrate into ischemic tissues [8, 9]. There, they can adopt endothelial characteristics thus, contributing to neovascularization [10]. This is at least partially facilitated by direct incorporation into newly formed capillaries [10, 11]. Furthermore, EPC stimulate endogenous angiogenesis by secreting a variety of angiogenic growth factors [7]. They also release factors that directly stimulate keratinocyte and fibroblast proliferation during wound healing [7]. Capillaries can be detected in granulation tissue by CD90 expression, that can be found on activated microvascular endothelial cells and CD31 expression, that is a linage marker for vascular endothelial cells and is also involved in angiogenesis [12].

Homing of EPC to injured tissues is triggered by VEGF and SDF-1α that is mainly released by platelets [13–15]. For this reason, EPC express vascular endothelial growth factor receptor 2 (VEGFR2) and stromal cell-derived factor 1α (SDF-1α) receptor CXCR4 [13, 14]. It has been shown that under pathological conditions like diabetes, homing of EPC by SDF-1α expression is impaired [16].

Previous studies by us, and others demonstrated that delivering EPC to wounds, either by local injection or systemically significantly accelerates healing and promotes neovascularization in granulation tissues as well as in bone defects [17–19]. Suh et al. could also show an improvement in dermal wound healing after local transplantation of human blood derived early EPC into dermal wounds of immunodeficient nude mice [17].

Still number and purity of EPC that can be harvested from bone marrow or blood is limited [20]. Considering this limitation, we wanted to investigate in the following study whether a lower number of EPC locally administered has the same effect on wound healing as a higher number of these cells systemically applied. For this reason, we used a standardized wound model in hairless mice and directly compared systemic versus local administration of cultivated EPC monitoring and epithelialization throughout the healing process as earlier described [21].

## Materials and methods

### Animals care and wound model

All procedures were performed in accordance with the guidelines set by German law for the care and use of laboratory animals. The experimental study was approved by the regulatory authorities (Regierungspräsidium Darmstadt) under the Ethic Approval Number v54-19c2015-F3/12.

Male homozygous hairless mice (SKH-1, 20–30 g, 8–12 weeks, Charles River Laboratories, Sulzfeld, Germany) were housed in separate cages in room temperature (24 °C), light (12 h/day) and airflow regulated rooms. They were fed a balanced rodent diet and water ad libitum.

All procedures were performed with the animals anesthetized with intraperitoneal injection (i.p.) of 100 µL solution containing 2.215 mg of ketamine and 0.175 mg of xylazine hydrochloride. After desinfecting the ears, mice were placed on a plexiglas platform with their ears extended on a microscope slide by placing three permanent loops (9–0, nylon) at opposite poles of their ears. Standardized, circular wounds (2.25 mm in diameter, 125 µm in depth) were created on the dorsum of the ears using a punch. Wounds were positioned between the ears’ anterior and middle principle neurovascular bundles. After the punch incision, a full thickness layer of skin within the punch was dissected away down to the underlying cartilage [21–25]. The day of wounding was designated as day 0.

Immediately after surgery, in the groups receiving systemic EPC, 2 × 10^6^ EPC (in 250 µL PBS) or PBS (250 µL) were injected into the tail vein [18]. In the groups receiving local EPC, 2 × 10^5^ EPC (in 30  µL PBS) or PBS (30 µL) alone was injected directly into the wound [17]. Wounds were covered with self-adhesive polyurethane foam dressing (Allevyn thin; Smith and Nephew Medical Ltd., Hull UK) and the entire ear was then covered with a bio-adhesive dressing (Opsite; Smith and Nephew Medical Ltd.) to protect the wound from contamination and mechanical irritation.

### EPC isolation and culture

EPC were isolated as in our previous work by density gradient centrifugation (20 min, 600 g) with Ficoll (1.077 g/mL, Biochrom, Berlin, Germany) from the spleen of homozygous hairless mice (SKH-1, 20–30 g, 8–12 weeks, Charles River Laboratories, Sulzfeld, Germany) after mechanically mincing using syringe plungers [18].

After isolation, total EPC (4 × 10^6^ cells, cell density 2 × 10^6^ cells/cm^2^) were cultured on fibronectin-coated (10 μg/mL; Sigma, Deisenhofen, Germany) 24-well plates maintained in 0.5 mL endothelial cell basal medium (EBM-2) supplemented with endothelial growth medium SingleQuots (EGM-2 MV; Clonetics, Cambrex, Walkersville, MD) at 37 °C, 5% CO_2_. Non-adherent cells were removed after 4 days and adherent cells were incubated in medium for another 24 h prior to initiation of the experiments.

To detect EPC in vivo in healing wounds, they (after 5 days of culture) were harvested by Accutase (PAA Laboratories, Pasching, Austria) for 10 min at 37 °C, 5% CO_2_, and 2 × 10^6^ and cells were re-suspended in 250 µL PBS. To detect EPC incorporation, EPC were pre-labeled with 2.5 µg/mL DiLDL in EBM-2 supplemented with 20% FCS for 1 h at 37 °C, 5% CO_2_, followed by harvest and administration.

Intravitally, EPC were visualized under a fluorescent microscope and pictures were merged with light microscope image.

### Measuring wound reepithelialization and closure

Epithelialization and EPC recruitment were directly visualized and measured using intra-vital microscopy and computerized planimetry. Microscopic area measurements were performed immediately after wounding and every second day thereafter up to complete wound closure. When epithelialization was near completion, the wounds were observed daily to determine the exact day each process was completed. Measurements were performed by placing anesthetized mice with the Plexiglas platform on the stage of an intra-vital microscope (Carl Zeiss, Oberkochen, Germany). The microscope images were captured with a low light camera (DXC-390P, 3CCD color video camera; Sony, Tokyo, Japan) and transmitted through a digital converter (ADVC-100; Canopus, Ruppach-Goldhausen, Germany) to a monitor. Photographic images were analysed by tracing the wound margin and calculating the area using ImageJ software (http://rsb.info.nih.gow/ij/download.html). The rate of wound closure was expressed as the ratio of the wounded area at each time point divided by the area of the original wound at day 0. The analysis was performed off-line in a blinded fashion by a different investigator not knowing the treatment each animal received.

### Measuring wound neovascularization and SDF-1α expression

Tissue samples were taken from the wound area at days 3, 6, 9 and 12 after wounding. The tissues were dehydrated by isopentane, embedded in TissueTek (Sakura Finetaek Europe, Zoeterwoude, Netherlands) and stored at − 80 °C for subsequent examination.

As capillaries can be detected in granulation tissue by CD90 expression and CD31 expression, wounds were stained for these markers as well as for SDF-1α [12].

For analysis, 6 µm thick wound sections, prepared as described above, were treated with acetone (− 20 °C, 10 min) and 0.1% hydrogen peroxidase to quench the endogenous peroxidase activity. Sections were stained with primary antibodies (Abcam, Cambridge, UK) directed against CD31 (1:100; ab7388), CD90 (1:150; ab3105), VEGF (1:100; ab1316) and SDF1α (1:100; ab25117) for 1 h at RT. Primary antibodies were detected by HRP-AEC (Abcam) staining according to the guidelines of the manufacturer. All sections were counterstained with hematoxylin and viewed at 100× magnification (Axio Observer; Carl Zeiss, Oberkochen, Germany). The microscope image was captured with a low light camera (AxioCam; Carl Zeiss) and digitized. Photographic images were analyzed by tracing the stained areas and calculating the area, using ImageJ software. The staining of each section was randomized to the mean value of the granulation tissues' area from all groups. The analysis was performed off-line in a blinded fashion by a different investigator not knowing the treatment each animal had received.

### Experimental groups

Animals were randomly allocated into four treatment groups (*n* = 10 per group):

PBS sys (control systemic) = Animals receiving systemic PBS (250 μL) alone.

PBS loc (control local) = Animals receiving local PBS (30 µL) alone.

EPC sys = Animals receiving systemic EPC and PBS (2 × 10^6^ cells in 250 μL PBS).

EPC loc = Animals receiving local EPC and PBS (2 × 10^5^ cells in 30 µL PBS).

For immunohistochemical analysis of the wounds were performed on day 3, 6, 9 and 12 after wound creation (*n* = 8 per group). They were treated in the same manner as the in vivo groups.

### Statistical analysis

Data are presented as the mean ± standard deviation (SD). Statistical evaluation was performed with Kruskal–Wallis test followed by a Dunn post hoc test using a Bonferroni–Holm adjustment with Bias 10.0. Values of *p* < 0.05 were considered statistically significant. The number of samples examined is indicated by *n*.

## Results

### Wound reepithelialization and closure

After EPC transplantation, we evaluated wound epithelialization and closure in animals treated with EPC locally and systemically.

**Fig. 1:**
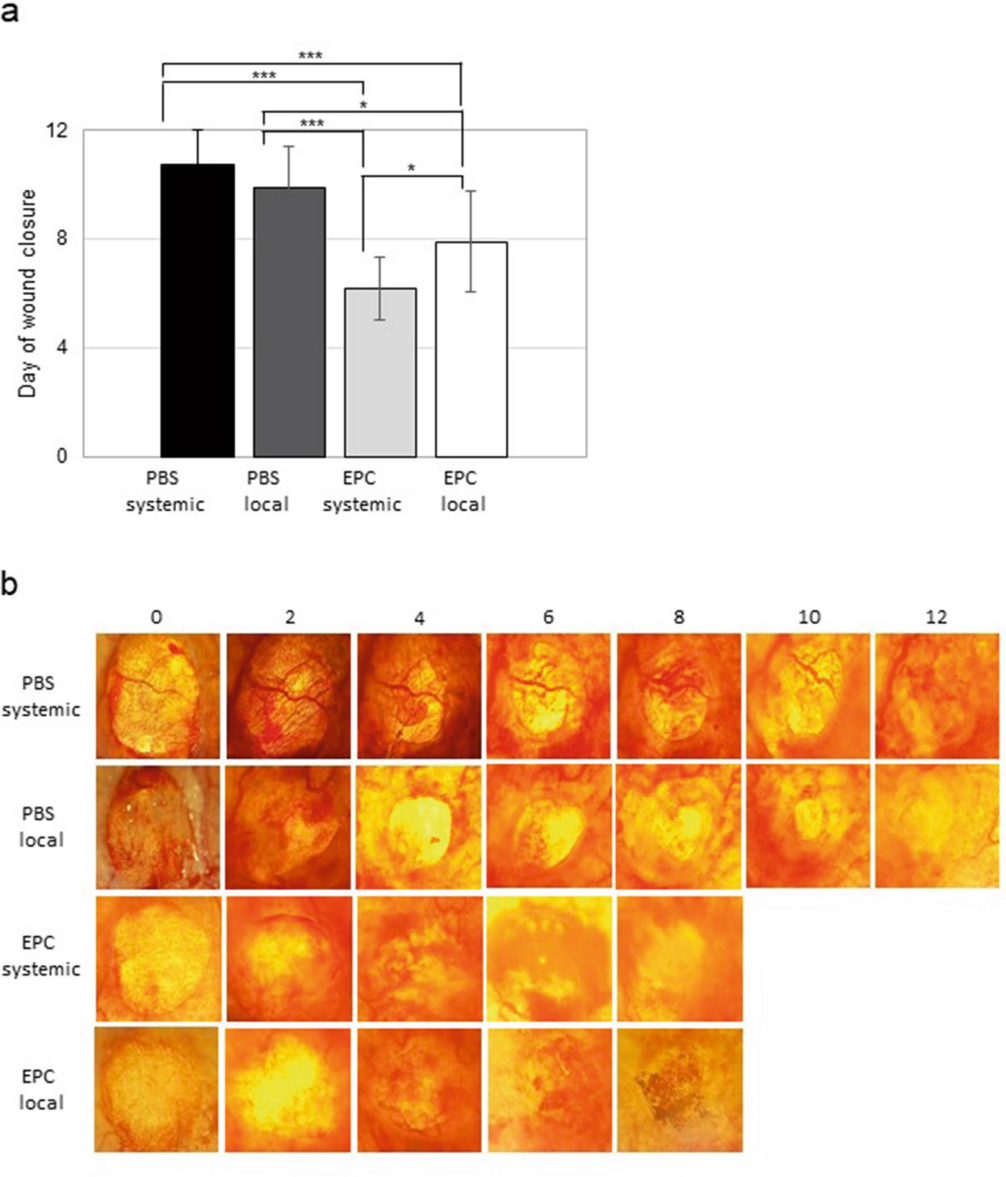
Day of wound closure. **a** Comparison between local and systemic PBS as well as systemic and local EPC application (data is shown as mean ± SD; *n* = 10; *p < 0.05, ***p < 0.001); **b** representative pictures of wounds throughout the wound healing process after systemic and local PBS treatment as well as systemic and local EPC application

**Fig. 2:**
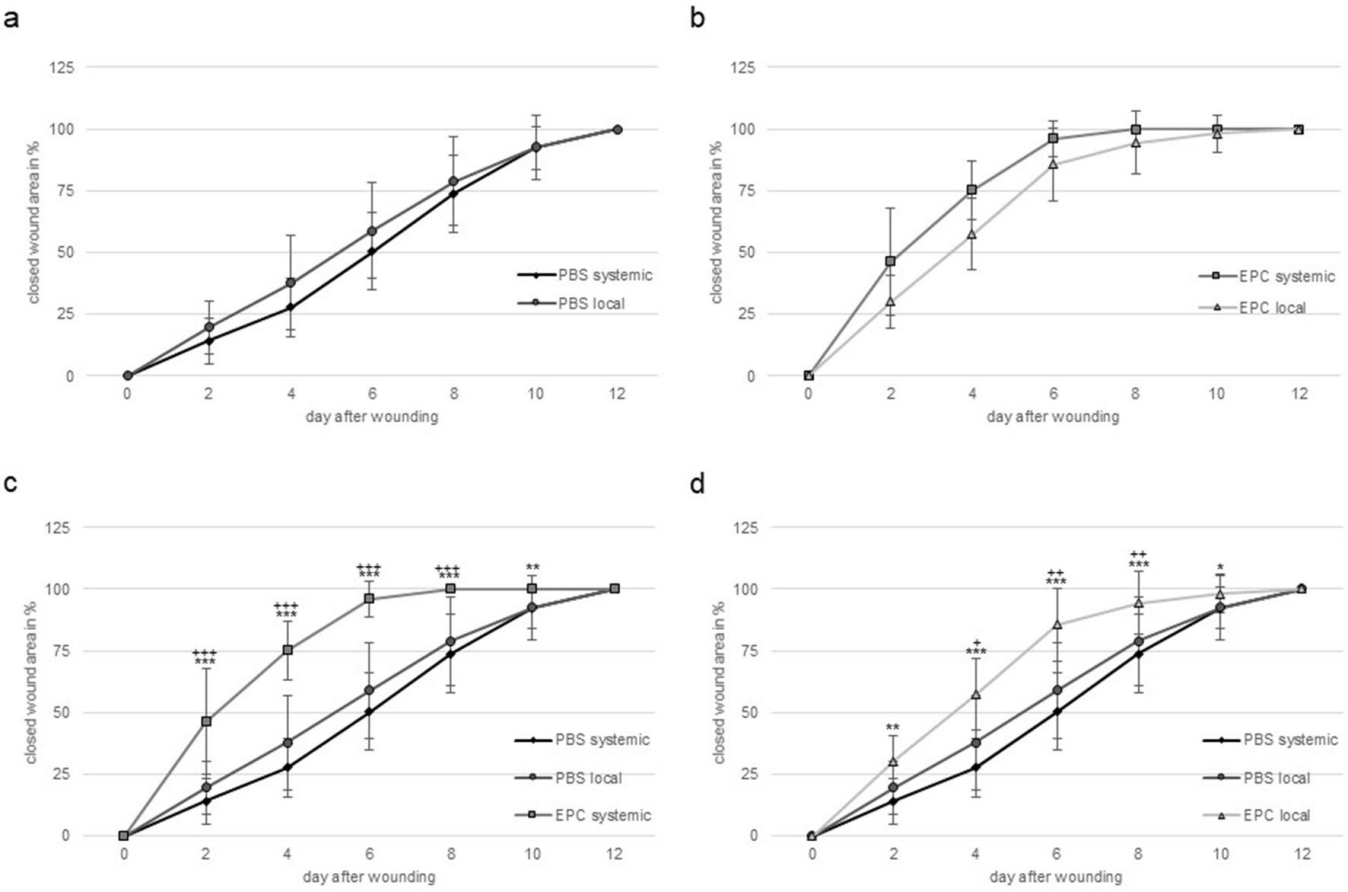
Percentage of closed wound area from ay 0–12. **a** comparison between systemic and local PBS treatment; **b** comparison between systemic and local EPC treatment; **c** comparison between both PBS treatment groups and systemic EPC; **d** comparison between systemic and local PBS to local EPC (data is shown as mean ± SD; *n* = 10). *p < 0.05, **p < 0.01, ***p < 0.001 comparison EPC systemic or local with PBS systemic; ^+^*p* < 0.05, ^++^*p* < 0.01, ^+++^*p* < 0.001 comparison EPC systemic or local with PBS local

Wounds receiving EPC systemically and locally closed significantly (*p* < 0.05) faster than PBS treated controls (PBS systemic day 10.75 ± 1.25 SD; PBS local day 9.90 ± 1.48; EPC systemic day 6.20 ± 1.15 SD; EPC local day 7.90 ± 1.83 SD; Fig. 2a). There also was a significant difference in closure rate between wounds treated with EPC locally versus those treated systemically (*p* < 0.05; Fig. 1a). No significant difference in day of wound closure was observed comparing the two different PBS treatment groups (Fig. 1a).

These findings are supported by rate of reepithelialization. Systemic and local EPC treatment displayed significantly faster coverage of wound area, for systemic treatment from day 2 to day 10 compared to both PBS controls and for local treatment from day 2 to day 10 compared to PBS systemic as well as from day 4 to day 8 compared to PBS local (Fig. 2c, d). The two PBS treatment groups showed a similar reepithelialization rate (Fig. 2a). Whereas systemic transplantation of EPC seemed to have a slight beneficial effect on wound closure, compared to local treatment, though this finding was not significant (Fig. 2b).

### Wound neovascularization

**Fig. 3:**
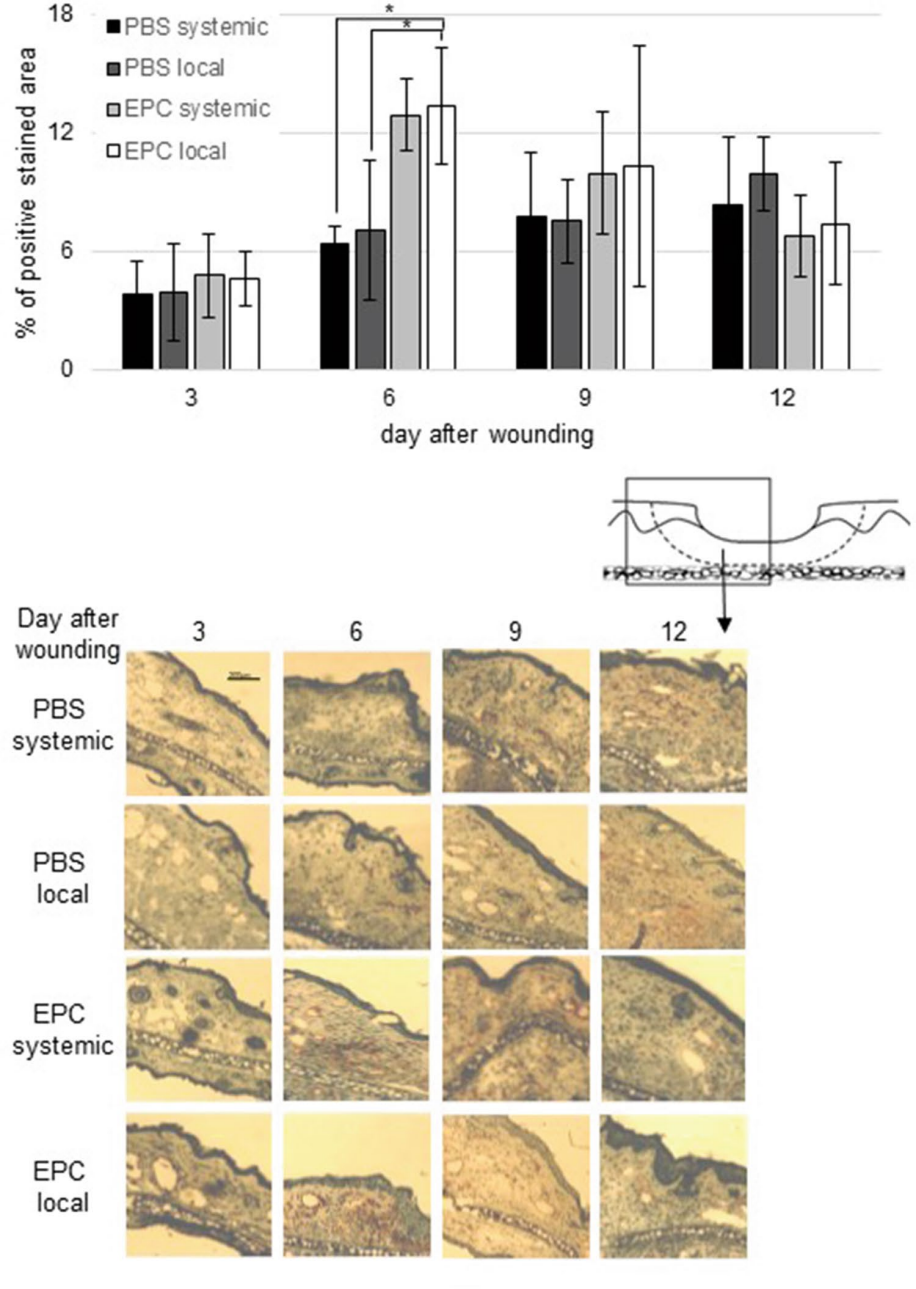
Percentage of CD31 positive wound area on day 3, 6, 9, and 12 after wounding of PBS- and EPC-treated wounds. Below representative pictures of immunohistological staining (data is shown as mean ± SD; *n* = 6). *p < 0.05

To investigate the effect of local and systemic transplanted EPC on neovascularization at the site of injury, capillary density of wound sections was measured by immunostaining for CD31. CD31 was significantly higher in animals whose wounds received local EPC than in both PBS controls on days 6 (Fig. 3). There also seemed to be an elevation in CD31 after systemic EPC transplantation on day 6, though this change was not significant (Fig. 3). Comparing the data of local versus systemic effect of EPC transplantation on CD31 expression no significant difference was noticed (Fig. 3).

**Fig. 4:**
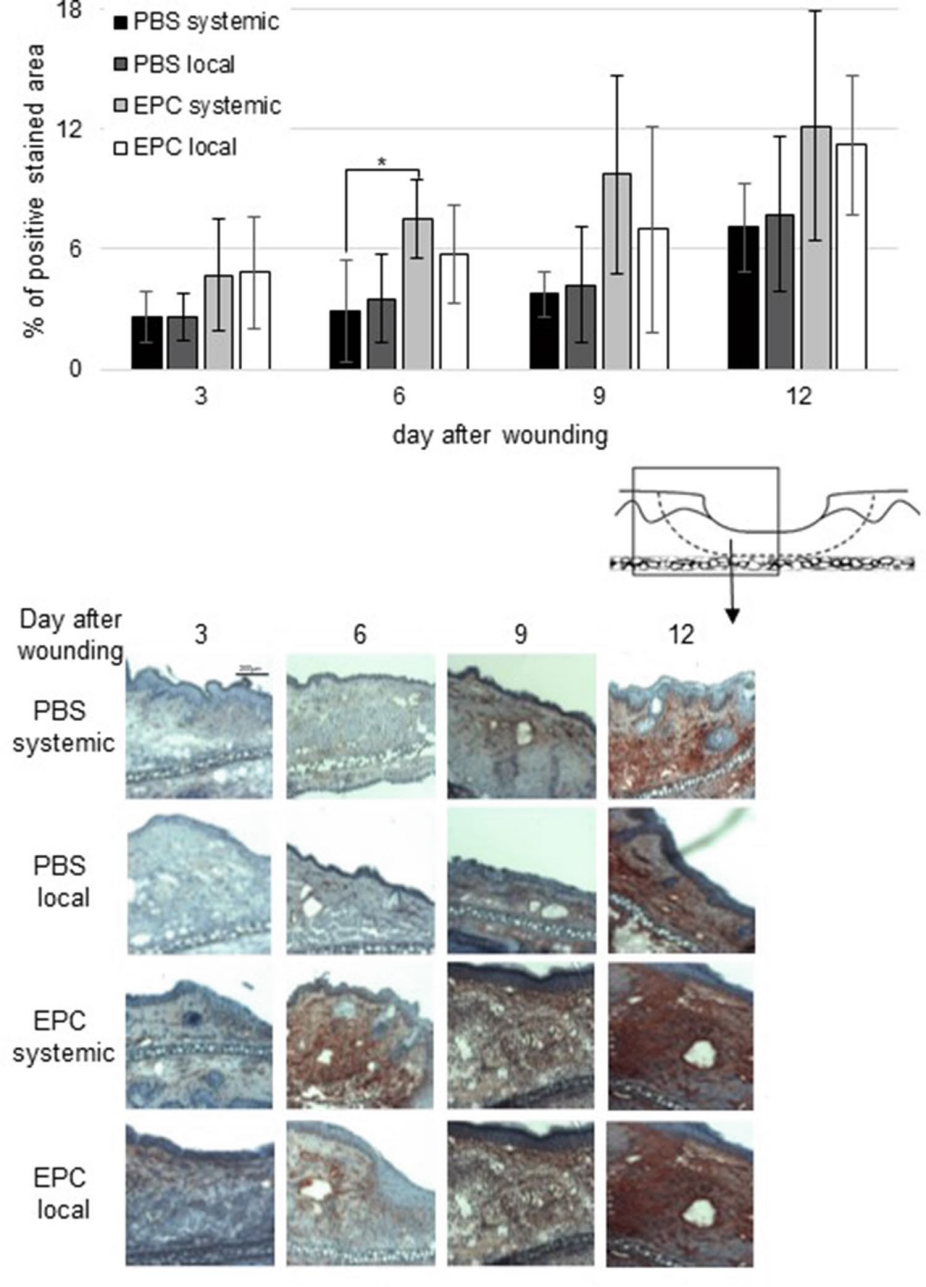
Percentage of CD90-positive wound area on day 3, 6, 9, and 12 after wounding of PBS-and EPC-treated wounds. Below representative pictures of immunohistological staining (data are shown as mean ± SD; *n* = 6). *p < 0.05

CD90 expression tended to be raised by local as well as systemic transplantation of EPC though this finding was only significant on day 6 for systemic transplantation compared to PBS systemic (Fig. 4). Systemic EPC application also elevated CD90 expression compared to local treatment on day 6, 9 and 12 though this also was not significant.

**Fig. 5:**
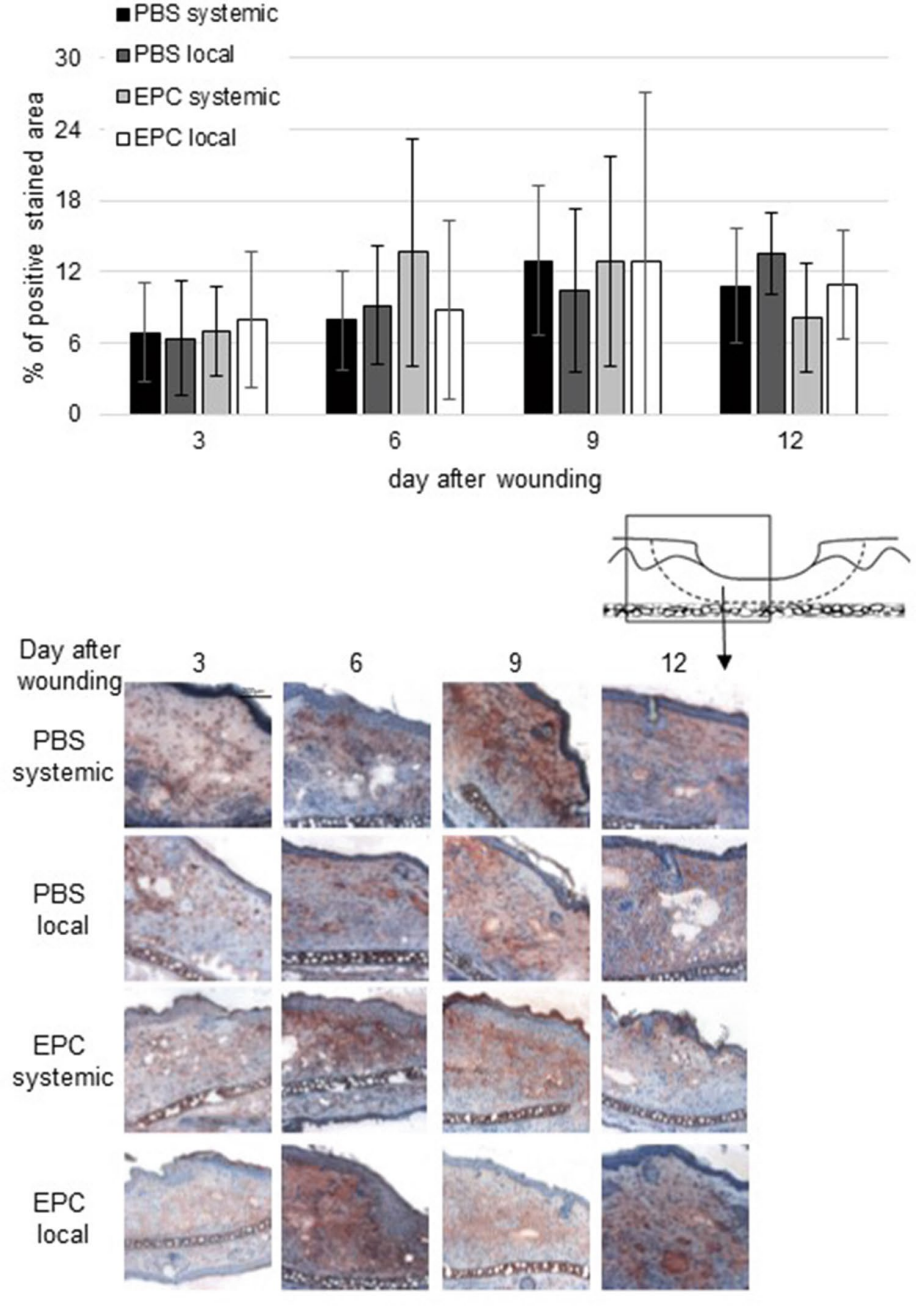
Percentage of VEGF-positive wound area on day 3, 6, 9, and 12 after wounding of PBS- and EPC-treated wounds. Below representative pictures of immunohistological staining (data are shown as mean ± SD; *n* = 6)

Regarding VEGF expression in wounds, surprisingly, there was no significant difference observed comparing all treatment groups (Fig. 5).

### EPC migration and homing

#### EPC accumulation at wound margins (measured by intra-vital microscopy)

**Fig. 6:**
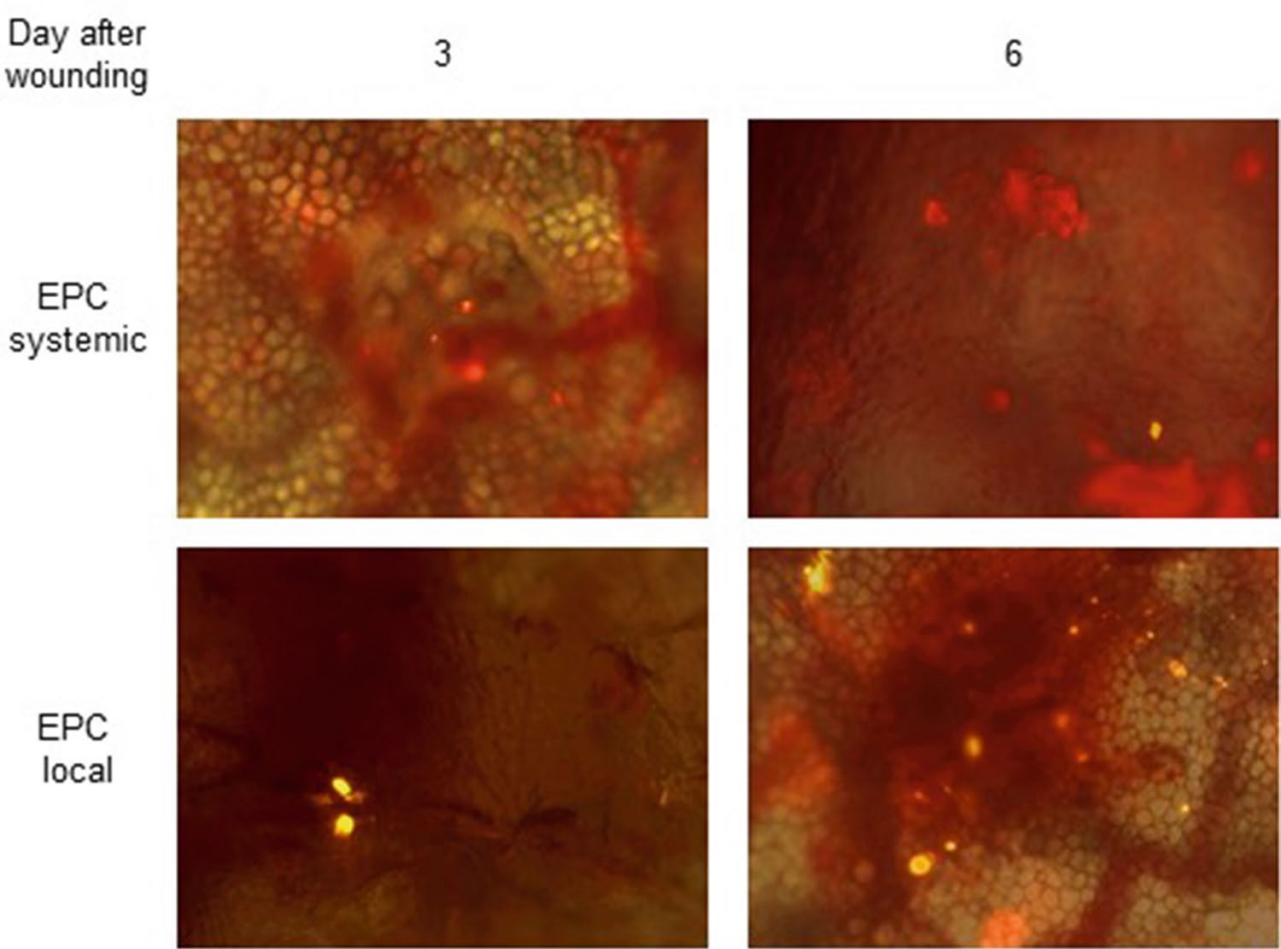
Representative intra-vital pictures of pre-labeled DiLDL EPC at the wound margin on day 3 and 6 after wounding

EPC were pre-labeled with red fluorescent DiLDL prior to local or systemic administration to assess EPC homing to, and survival in the wound. In animals receiving local and systemic EPC, fluorescent-labeled EPC were observed around the wound margins at 3 and 6 days after wounding using intra-vital microscopy (Fig. 6).

**Fig. 7:**
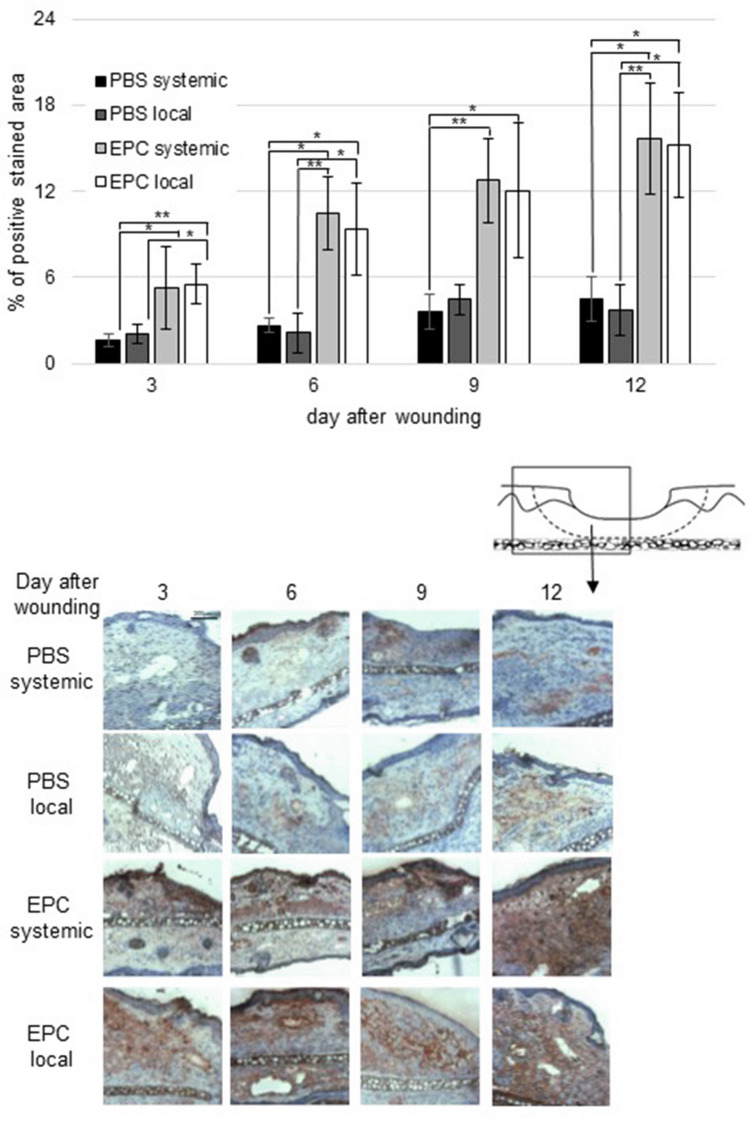
Percentage of SDF-1α-positive wound area on day 3, 6, 9, and 12 after wounding of PBS- and EPC-treated wounds. Below representative pictures of immunohistological staining (data are shown as mean ± SD; *n* = 6). *p < 0.05; **p < 0.01

As an important homing factor for EPC, SDF-1α expression was significantly upregulated in the wound on all days investigated receiving local and systemic EPC when compared to PBS systemic application (Fig. 7). There was also a significant elevation of SDF-1α on day 6 and 12 comparing systemic EPC application to PBS local as well as on day 3, 6 and 12 comparing local EPC application to PBS local (Fig. 7). No significant difference was noticed between the two EPC treatment groups.

## Discussion

The aim of this study was to determine the effect of local versus systemic EPC treatment on dermal wound epithelialization, neovascularization and closure. With the unique model used, we are able to directly monitor reepithelialization as there is no significant wound contraction as the dermis is connected to the underlying cartilage, thus mimicking the process more accurately to wound healing in humans [21, 26].

EPC are already known to enhance tissue regeneration and wound healing [5, 18, 27]. In response to ischemia or vascular injury, they are released into peripheral circulation. They migrate to damaged tissues and promote endothelial healing and angiogenesis.

We could confirm the ability of EPC to enhance wound healing in this study as it has already been shown by several groups before [17, 18, 28]. Furthermore, we could demonstrate that local injection of EPC in wounds had almost the same effect as systemic i.v. treatment on wound closure and epithelialization (Figs. 1 and 2). This finding is remarkable, since locally treated wounds received a tenfold lower amount of EPC then their systemically treated counterparts.

Although it is known that delivering cells directly into damaged tissue causes cell loss compared to systemic application, Bonaros et al. and Reinecke et al. also reported that local delivery of cardiomyocytes directly into the heart resulted in a significantly greater number of cells in the infarcted regions [29, 30]. Regarding this, the effect of local EPC transplantation is even more remarkable as we only applied the cells around the wound margin and not in the local vessels for treatment. Nevertheless, wound healing was ameliorated by local as well as systemic delivery. Thus, our findings show that the cellular effects seem not only to be connected to EPC delivery in local vessels or through blood flow.

Furthermore, it has already been shown that systemic transplantation requires a higher amount of cells due to a poor distribution and low cell survival [31]. 90% of systemic transplanted EPC are entrapped in undesired organs including the liver, spleen and kidney when cells were intravenously injected [31]. The positive effects of local EPC transplantation and our conclusions are supported by the findings of Kim et al. They demonstrated that delivering of EPC directly into diabetic wounds not only enhanced neovascularization but also activated the proliferation of local keratinocytes and fibroblasts [32].

In cardiovascular disease models, circulating EPC have been shown to preferentially home to ischemic tissue where they are directly incorporated into vessel walls [8]. We could demonstrate the presence of the systemically as well as locally transplanted pre-labeled EPC at the wound area in our model by intra-vital microscopy. Incorporated endothelial progenitor cells are able to promote neovascularization and cardiac regeneration by releasing growth factors, which act in a paracrine manner to support local angiogenesis and mobilize progenitor cells residing in the local tissue [33]. They also promote recruitment of monocytes and macrophages which furthermore support neovascularization [17, 34].

In our hands, local application of EPC enhanced the expression of CD31 at the wound margin on day 6 after wounding comparing the control groups (Fig. 3). Systemic treatment also seemed to enhance CD31 expression on day 6 though this finding was not significant. CD31 expression is used to evaluate angiogenesis that in turn leads to a faster wound closure [35].

Furthermore, we demonstrated in our study an increase in CD90 expression from day 3 to day 12 after local and systemic EPC transplantation, though this finding was only significant for systemic treatment compared to systemic PBS injection on day 6 after wounding. CD90 is known as a versatile modulator of signalling affecting cellular adhesion, proliferation, survival and cytokine/growth factor responses [36]. CD90 is used to monitor vascular density in granulation tissue [18]. Thus, elevated expression of CD31 and CD90 by local as well as systemic EPC transplantation marks enhancement of local vascularization.

In contrast to enhanced expression of CD31 and CD90, there was no significant difference in VEGF expression in wounds after systemic or local EPC transplantation compared to control groups. VEGF is a potent angiogenic growth factor, which induces increased vascular permeability, proliferation, migration and recruitment of EPC from the bone marrow [33]. EPC attracted to wound site are known to incorporate directly into neovasculature and also augment angiogenesis through the secretion of VEGF [17]. Therefore, enhanced vascularization after local and systemic EPC application seemed to be independent of VEGF signalling as we found enhanced vascularization by CD31 and CD90 expression. This might be mainly explained by the fact that EPC can be directly incorporated into the neovasculature during the repair [17]. Furthermore, they promote endogenous angiogenesis by secreting angiogenic growth factors at EPC-incorporated foci which in turn contributes to the development of host-derived neovessels [17]. Another VGF-independent way of enhancing neovascularization by EPC is the paracrine modulation of endothelial cells by exosomes [37]. Therefore, we propose that the positive effects of local EPC transplantation on wound healing is partly due to paracrine effects of the EPC and an improved cell–cell interaction between EPC and local endothelial cells.

We also found a significant upregulation of SDF-1α at wound margins on all days investigated after local and systemic EPC transplantation (Fig. 7). SDF-1α is produced by tissue ischemia or in response to vessel damage [2]. SDF-1α promotes trans-endothelial migration of progenitor cells toward vascular lesions and in the endothelial damage site, which in turn exposes specific adhesion molecules [38]. Furthermore, it can also enhance keratinocyte proliferation and migration in vitro helping the process of reepithelialization [39]. Thus, upregulation of SDF-1α contributes to amelioration of wound healing by local and systemic EPC treatment.

Also under pathological conditions as ischemia or diabetes, transplantation of EPC has been found to be a promising treatment strategy [28, 40]. Therefore, it will be interesting to investigate whether local treatment can reduce the number of EPC needed in disease models as well to pave the way for clinical trials.

## Conclusion

Our data suggest that local delivery of EPC is an attractive therapeutic approach for treating acute and chronic dermal wounds. Moreover, local treatment significantly reduces the amount of autologous EPC needed in comparison to systemic application.

## Data Availability

The data sets used and analyzed in the current study are available from the corresponding author on reasonable request.
